# Amine‐AlH_3_ Adducts as Energetic Materials for a New Generation of Solid Fuels

**DOI:** 10.1002/chem.202501163

**Published:** 2025-06-26

**Authors:** Xiaoran Liu, Jochen Ortmeyer, Alexander Bodach, Hilke Petersen, Michael Felderhoff

**Affiliations:** ^1^ Max‐Planck‐Institut für Kohlenforschung Kaiser‐Wilhelm‐Platz 1 45470 Mülheim an der Ruhr Germany

**Keywords:** aluminum hydride, combustion, energetic materials, hypergolic properties, mechanochemistry

## Abstract

Energetic materials, mainly propellants, explosives, and pyrotechnics, are crucial in various civilian applications, such as fuels for rockets and spacecraft. Current energetic fuels rely on highly toxic and polluting ammonium perchlorate (AP) or carcinogenic hydrazine derivatives, encouraging the search for greener and safer substitutes. This work demonstrates the first use of amine‐AlH_3_ adducts as potential solid fuels with promising hypergolic properties, high energy content, and without toxic derivatives. The herein presented four amine‐AlH_3_ adducts, AlH_3_ coordinated with quinuclidine (Quin), triethylenediamine (TEDA), hexamethylenetetramine (HMTA), and tetraazatricyclododecane (TATD), illustrate a unique strategy to create new solid fuels by combining AlH_3_ with an energy‐rich nitrogen‐containing molecule. The crystal structures of new compounds ([HMTA‐AlH_3_]_n_ and [TATD‐AlH_3_]_n_) are determined from powder X‐ray diffraction data. Differential scanning calorimetry‐thermogravimetric analysis (DSC‐TGA) of the samples combined with mass spectrometry (MS) evidence high thermal stability. The ultrashort ignition delays as low as one ms show an excellent hypergolic response. Four amine‐AlH_3_ adducts have higher combustion heat (> 29 kJ g^−1^) than conventional hydrazine fuels (19.5 kJ g^−1^). Finally, the successful mechanochemical syntheses of Quin_2_AlH_3_ and [HMTA‐AlH_3_]_n_ are introduced, showcasing a green chemistry approach to energetic materials.

## Graphical abstract

New amine‐AlH₃ adducts offer a more environmentally friendly, high‐energy alternative to conventional rocket propellants, and exhibit ultra‐fast ignition. They can be synthesized by mechanochemical method under hydrogen pressure starting from aluminum metal directly or by wet chemical processes with AlH_3_.

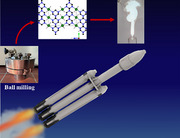

## 1 Introduction

Energetic materials, such as propellants, explosives, and pyrotechnics, have been increasingly crucial for civilian and space exploration applications.^[^ ^1^ ^]^ Propellants are an essential class of energetic materials, especially for aerospace applications.^[^ ^2^ ^]^. The typical solid propellant system consists of ammonium perchlorate (AP) as the oxidizer, aluminum as the fuel/reducer, and hydroxyl‐terminated polybutadiene (HTPB) as the binder. It is used widely in civil pyrotechnics, large booster engines in space shuttles, and heavy‐lift launchers.^[^ ^3^ ^]^ However, the combustion of AP results in the release of hydrochloric acid, posing environmental and health risks such as ozone depletion, acid rain formation, and thyroid dysfunction.^[^ ^4^ ^]^.With the rapid development of space exploration and severe potential ecological consequences caused by using standard propellant components, the investigation for new rocket propulsion with even higher performance and environmentally and economically acceptable compositions is required.^[^ ^5^ ^]^ Current research in this area is focused on developing new green alternatives with preserved performance, improved environmental acceptability, and more sustainable synthesis methods.^[^ ^6^ ^]^ The EU Horizon 2020 research and innovation program granted the GRAIL project, which aims to study a new system using a mixture of ammonium dinitramide (AND)/ammonium nitrate (AN), aluminum powder, and a polymer binder to replace AP‐based systems. The project investigated high‐energy fuels such as aluminum and aluminum hydride (AlH_3_) to achieve high performance.^[^ ^7^ ^]^

Aluminum is the most common cost‐effective metal fuel for solid propellants to improve the energy density and specific impulse.^[^ ^8^, ^9^, ^10^ ^]^ Aluminum particles ejected from the propellant surface will melt (∼ 600 °C) and then react in the gas flow in a diffusion flame, producing aluminum oxide (Al_2_O_3_).^[^ ^11^ ^]^ However, there is a vast difference between the combustion surface temperatures of the propellant (500 ^–^ 700 °C) and the ignition temperature of Al powder (1400 ^–^ 2100 °C), depending on particle sizes and other factors, such as additives (e.g., NaCl reduces the combustion temperature).^[^ ^12^, ^13^, ^14^ ^]^ Aluminum boils and coalesces before reaching the ignition temperature due to inflammation, which leads to incomplete burning and large agglomerations of aluminum powder, thereby slowing the gas flow rates, which is called two‐phase flow loss. The two‐phase flow losses can cause a substantial reduction in the delivered specific impulse, and large Al_2_O_3_ residue can also cause slag accumulation.^[^ ^11^, ^14^ ^]^

To address this issue, AlH_3_ has been proposed as a propellant additive to replace aluminum.^[^ ^8^, ^15^, ^16^, ^17^ ^]^ AlH_3_ has a high energy density (∼ 41.7 kJ g^−1^),^[^ ^18^ ^]^ with 148 g L^−1^ volumetric hydrogen storage capacity – twice as high as liquid hydrogen.^[^ ^19^, ^20^ ^]^ AlH_3_ is an ideal fuel supplement for solid rocket propellants because it can significantly increase the specific impulse of the propulsion system while at the same time reducing the flame temperature due to its high heat release and gas‐volume contribution, as well as rapid dehydrogenation at mild temperatures.^[^ ^15^, ^16^, ^17^ ^]^ For example, thermochemical calculation using the NASA CEA chemical equilibrium code indicated that replacing aluminum with AlH_3_ in a typical composite solid rocket propellant (AP/HTPB/Al) increased the specific impulse by 7 ∼ 8%.^[^ ^21^ ^]^ Furthermore, at 90% solids loading, the flame temperature of the highest‐performing galvanized propellant was 15% lower than that of the highest‐performing aluminized propellant. The reduced flame temperature could alleviate two‐phase flow losses and reduce the thermal protection of a given rocket motor.^[^ ^21^ ^]^

Despite its potential benefits, manufacturing challenges, poor stability, and reactivity with moisture have hindered the widespread adoption of AlH_3_.^[^ ^22^, ^23^ ^]^ From the ‘60s’, interest in using AlH_3_ in rocket propellant inspired many efforts to synthesize air‐stable AlH_3_. Numerous studies have focused on stabilizing AlH_3_ through surface passivation, for example, surface oxidation and coating methods.^[^ ^24^, ^25^, ^26^, ^27^, ^28^, ^29^, ^30^, ^31^, ^32^, ^33^ ^]^ For instance, Petrie et al.^[^ ^32^ ^]^ treated AlH_3_ with acidic solutions (e.g., aqueous HCl), and the resulting product improved stability over an extended period. Kempa et al.^[^ ^34^ ^]^ reported that AlH_3_ coated with aluminum‐hydroxide clusters exhibits remarkable stability, which remains chemically stable even when stored underwater. Cortes et al.^[^ ^35^ ^]^ used ball milling to physically combine AlH_3_ with a triammonium salt of aurin tricarboxylic acid, and the product has a high resistance to air, moisture, and liquid water while maintaining reasonable hydrogen storage and release kinetics. Shi et al.^[^ ^36^ ^]^ modified the surface of AlH_3_ by a coupling agent, vinyltrimethoxysilane (A171), and the AlH_3_@A171 composite showed enhanced performance in terms of thermal stability and hygroscopic properties. It is worth pointing out that the obtained AlH_3_@A171 exhibited an increased activation energy after stabilization by coating with A171.

However, besides its application in propellant, AlH_3_ is also widely studied as a hydrogen storage medium, in which AlH_3_‐adducts, such as AlH_3_‐amine adducts, are investigated as intermediates to hydrogenate aluminum. Ashby^[^ ^37^ ^]^ in the 1960s synthesized triethylenediamine alane (C_6_H_12_N_2_·AlH_3_) directly from activated aluminum in the presence of triethylenediamine (TEDA) at 70 °C and 345 bar hydrogen pressure for 6 hours in tetrahydrofuran. Later on, Murib et al.^[^ ^38^ ^]^ used various tertiary amines to form amine alane adducts as an intermediate to obtain AlH_3_. The most recent efforts are mainly from Graetz et al.,^[^ ^24^, ^25^, ^26^, ^27^, ^28^, ^29^ ^]^ who synthesized AlH_3_‐dimethylethylamine, AlH_3_‐triethylenediamine, Nalkylpyrrolidine‐AlH_3_, AlH_3_‐trimethylamine, bis(quinuclidine) alane. All of the syntheses were performed in a 300 mL stainless‐steel stirred reactor loaded with Ti catalyzed Al (Al*), and amines (dimethylethylamine/TEDA/ *N*‐alkylpyrrolidine/trimethylamine/quinuclidine), and the solvents (tetrahydrofuran, toluene, or diethyl ether, depending on the reactions, different solvents are used) under hydrogen pressure, then by removing amines from systems, AlH_3_ was generated. Recently, Ortmeyer et al.^[^ ^39^ ^]^ synthesized triethylenediamine [TEDA^–^AlH_3_]_n_ adduct by both wet chemical methods and mechanochemical procedures (ball milling). [TEDA^–^AlH_3_]_n_ shows enhanced stability compared to AlH_3_, which makes it promising for practical application in energy fields.

In this paper, we describe the synthesis and characterization of two new amine^–^AlH_3_ compounds: hexamethylenetetramine‐AlH_3_ ([HMTA^–^AlH_3_]_n_) and tetraazatricyclododecane^–^AlH_3_ ([TATD^–^AlH_3_]_n_). Their crystal structures have been determined from powder X‐ray diffraction (PXRD) data. To verify the application of amine‐AlH_3_ adducts for fuels, we have investigated the hypergolic and combustion properties of several amine‐AlH_3_ adducts synthesized, including Quin_2_AlH_3_, [TEDA^–^AlH_3_]_n_, [HMTA^–^AlH_3_]_n_, and [TATD^–^AlH_3_]_n_. Differential scanning calorimetry^–^thermogravimetric analysis^–^mass spectrometry (DSC^–^TGA^–^MS) was used to compare their stability and decomposition behaviors. Additionally, the possibilities of synthesizing those compounds by the mechanochemical method were demonstrated, which could synthesize the desired products without solvents.

All amines used in this work are tertiary amines that are bound in 3D structures. The free electron pairs of the nitrogen atoms therefore have a high nucleophilicity and can form stable compounds to electron‐deficient molecules such as AlH_3_. In the case of secondary or primary amines, no stable AlH_3_ adducts are formed. All AlH_3_‐N adducts with primary or secondary amines are normally stabilized by the elimination of hydrogen or hydrocarbons.

## 2 Results and Discussion

### 2.1 Crystal Structure and Thermal Stability

**Figure 1:**
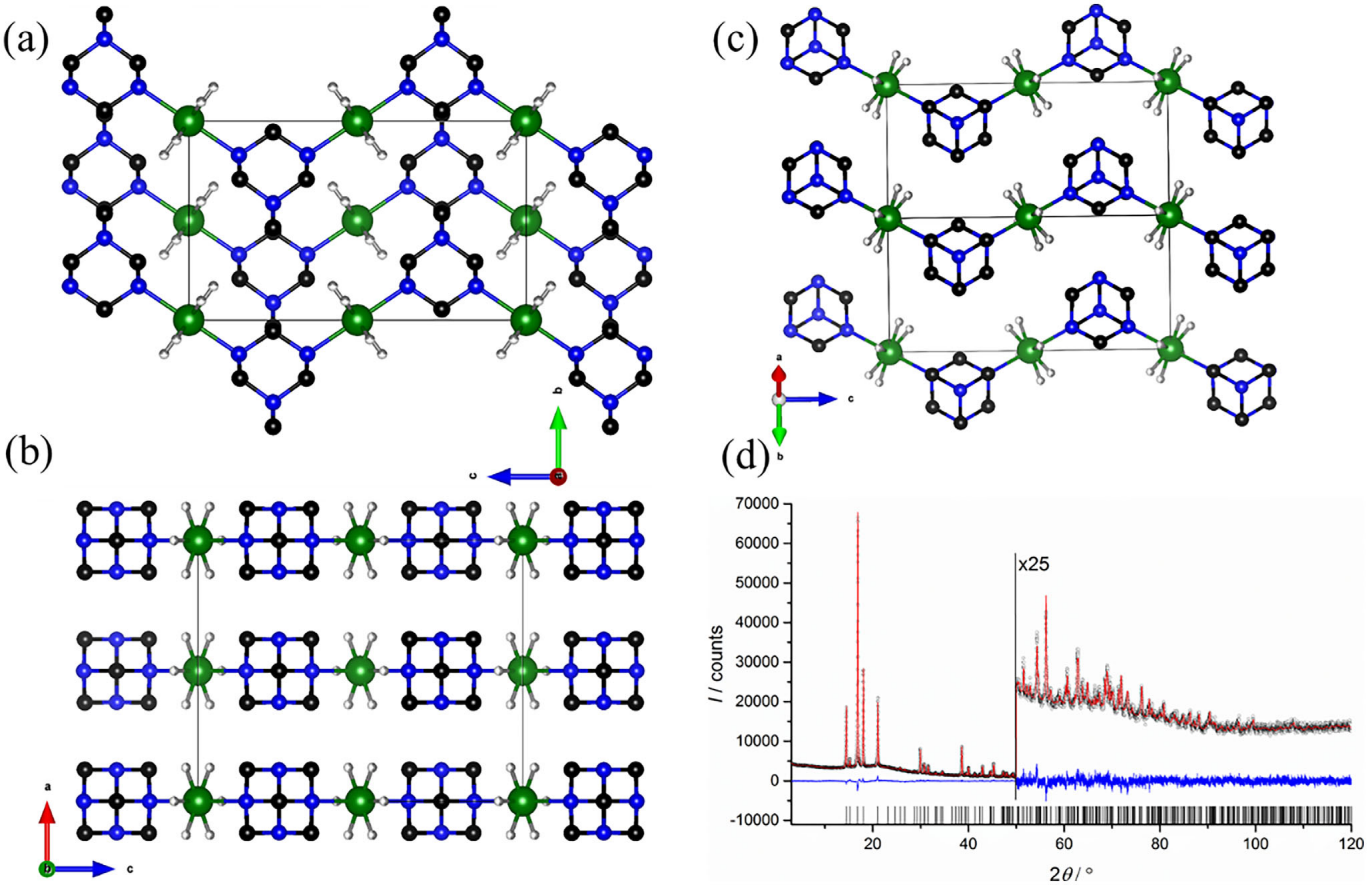
The packing motif of polymeric [HMTA–AlH_3_]_n_ (green: aluminum, blue: nitrogen, black: carbon, white: hydrogen‐disordered with 0.5 occupancy) depicted along the *a* axis a), *b* axis b), and *c* axis c), and the related Rietveld plot (d; black circles: measured intensities, red line: calculated intensities, blue line: difference curve, tick marks: positions of Bragg reflections). (Carbon‐bound hydrogen atoms were omitted for clarity.).

**Figure 2:**
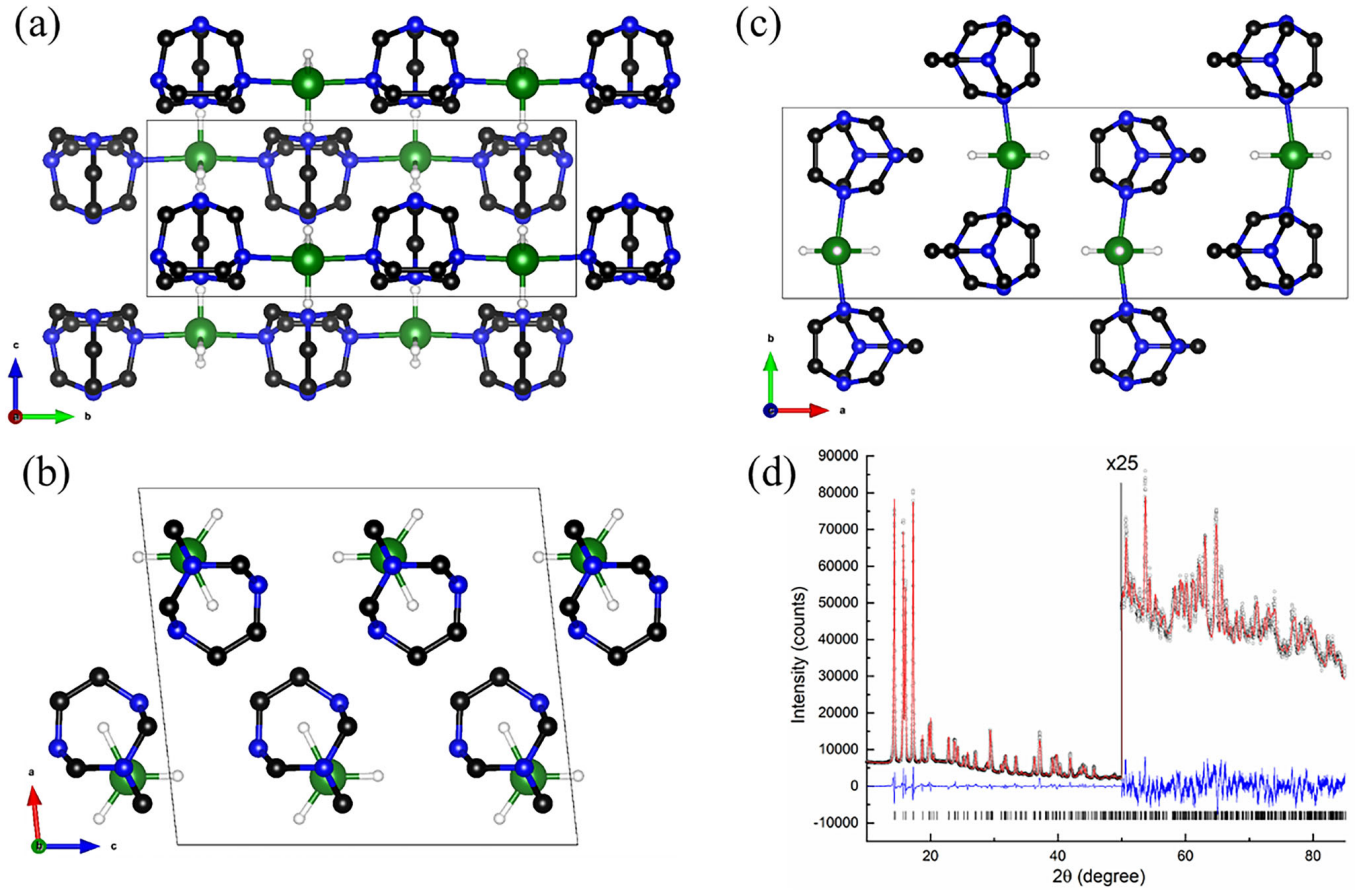
The packing motif of polymeric [TATD–AlH_3_]_n_ (green: aluminum, blue: nitrogen, black: carbon, white: hydrogen) depicted along the *a* axis a), *b* axis b), and *c* axis c), and the related Rietveld plot (d; black circles: measured intensities, red line: calculated intensities, blue line: difference curve, tick marks: positions of Bragg reflections). (Carbon‐bound hydrogen atoms were omitted for clarity.).

The crystal structures of the new compounds were determined from PXRD data obtained from the crystalline samples synthesized by the wet chemical method. Their respective IR spectra were used as a complementary method to validate the results and are reported in the Experimental Section, and the IR spectra are shown in Figure S1. The reactions of HMTA with AlH_3_ and TATD with AlH_3_ under the given conditions yielded products with polymeric structures similar to [TEDA^–^AlH_3_]_n._^[^ ^39^ ^]^ [HMTA–AlH_3_]_n_ crystallizes in an orthorhombic centrosymmetric space group *Cmcm* (no. 63) with *a* = 9.8151(1) Å, *b* = 7.2085(1) Å, *c* = 12.2019(1) Å, *α* = *β* = *γ* = 90°. The aluminum atom is penta‐coordinated by three hydrogen atoms in the basal plane and two equivalent nitrogen atoms in capping positions to form a nearly ideal (rotationally disordered) trigonal bipyramidal structure (Figure 1). The obtained structure models were refined via the Rietveld method,^[^ ^40^ ^]^ resulting in a good agreement (Figure 1d; *R*_wp_ = 2.74 %). The aluminum atom is located on the 2*/m* symmetry Wyckoff position *4a* at the origin. In contrast, the carbon atom is located on the *m*2*m* Wyckoff position 4*c*, and two nitrogen atoms and two carbon atoms occupy each position with *m* Wyckoff positions 8*g* and 8*f*. One hydrogen atom is bound to carbon, which is located on an *m* Wyckoff position 8*f*, while one carbon atom is located on an *m* Wyckoff position 8*g*. The three hydrogen atoms bound to aluminum are disordered on six positions with an occupancy of 0.5 each. Similarly to [HMTA–AlH_3_]_n_, the aluminum atom in [TATD–AlH_3_]_n_ is coordinated trigonally to three H atoms and the N1 atoms of the two neighboring TATD molecules, forming a trigonal bipyramidal structure. However, [TATD–AlH_3_]_n_ crystallized with a monoclinic unit cell in the space group *P*12_1_ */m*1 (no. 11) with *a* = 11.0539(1) Å, *b* = 7.4449(1) Å, *c* = 6.1596(1) Å, *β* = 96.199(1)°. The obtained structure models were refined via the Rietveld method,^[^ ^40^ ^]^ resulting in a good agreement (Figure 2d; *R*_wp_ = 3.56 %). In the resulting crystal structure, the Al and the connected H atoms occupy the Wyckoff positions 2*e* (Figure 2). N2, N3, C4, and C5 of the TATD building unit also occupy Wyckoff positions 2*e*. The residual atoms are sitting in general positions.

The Al─N bond length in the herein explored compounds decreases in the order: [TATD–AlH_3_]_n_ (*d*_Al_─_N_ = 2.291(6) Å) > [HMTA–AlH_3_]_n_ (*d*_Al_─_N_ = 2.190(2) Å) > [TEDA‐AlH_3_]_n_ (*d*_Al_─_N_ = 2.161(2) Å) > Quin_2_AlH_3_ (*d*_Al_─_N_ = 2.155(3) Å).^[^ ^22^ ^]^ The Al─H bond length in [HMTA‐AlH_3_]_n_ ranges from 1.515 Å to 1.520 Å, in [TATD‐AlH_3_]_n_ from 1.528 Å to 1.692 Å, in [TEDA‐AlH_3_]_n_ from 1.557 Å to 1.569 Å, and in Quin_2_AlH_3_ from 1.489 Å to 1.541 Å (Table 1). In all compounds, the Al atom is continuously coordinated with tertiary amines in a molar ratio of 1:1, even when tertiary amines, such as HMTA and TATD, have four nitrogen atoms in their structures. This could be caused by the decreased basicity of N atoms after coordinating with Al or increased steric hindrance.

**Table 1:** Bond lengths and decomposition temperatures of the herein explored compounds.

Compounds	*d*_Al─N_ [Å]	*d*_Al─H_ [Å]	Starting *T*_de_ [°C]	First Peak *T*_p_ [°C]
Quin_2_AlH_3_^[^ ^41^ ^]^	2.155(3)	1.489 – 1.541	75	113
[TEDA–AlH_3_]_n_^[^ ^39^ ^]^	2.161(2)	1.557 – 1.569	219	229
[HMTA–AlH_3_]_n_	2.190(2)	1.515 – 1.520	152	216
[TATD–AlH_3_]_n_	2.291(6)	1.528 – 1.692	112	159

(*T*_de_: decomposition temperature; *T*_p_: peak temperature)

**Figure 3:**
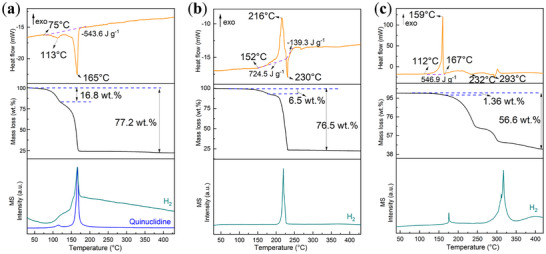
DSC (top)–TGA (middle)–MS (bottom) curves. a) Quin_2_AlH_3_, b) [HMTA‐AlH_3_]_n_, c) [TATD‐AlH_3_]_n_. (All samples are synthesized using the wet chemistry method.).

The DSC–TGA–MS results (Figure 3) show decreased starting decomposition temperature in the order of [TEDA–AlH_3_]_n_ (219 °C),^[^ ^39^ ^]^ [HMTA–AlH_3_]_n_ (152 °C), [TATD–AlH_3_]_n_ (112 °C), and Quin_2_AlH_3_ (75 °C), which is in conformity with the increased Al─N bond length, except for Quin_2_AlH_3_, as shown in Table 1. Quin_2_AlH_3_ decomposes in two steps (Figure 3a), each associated with an endothermic event. Notably, the onset decomposition temperature is approximately 75 °C, a significantly lower threshold than polymeric compounds such as [HMTA–AlH_3_]_n_ and [TATD–AlH_3_]_n_. Following this initial phase, we observed the concurrent release of hydrogen and the sublimation of quinuclidine, which was detected by MS analysis. In the first step, with the peak temperature at 113 °C, a weight loss of 16.8 wt.% is detected, more than the theoretical hydrogen content (1.2 wt.%) in the aluminum polyhedra of Quin_2_AlH_3_. Upon complete decomposition, a total weight loss of 77.2 wt.% is recorded, less than the theoretical value (89.31 wt.%) after decomposition, considering metallic aluminum as the final product after hydrogen release and quinuclidine sublimation. This disparity may be attributed to a plausible scenario wherein quinuclidine incompletely evaporates due to coordination forces with aluminum, potentially leading to nitrogen bonding on quinuclidine with aluminum and subsequent decomposition of alkaline groups.

The hydrogen release from [HMTA–AlH_3_]_n_ started at approximately 152 °C, reaching an exothermic peak temperature at 266 °C (Figure 3b). Notably, this peak temperature exceeded the onset decomposition temperature of HMTA (170 °C, Figure S2a), indicative of a sequential occurrence wherein dehydrogenation primarily happened in the presence of aluminum‐nitrogen bonding on HMTA, followed by subsequent HMTA decomposition. The TGA curve of [HMTA–AlH_3_]_n_ reveals an initial weight loss of approximately 6.5 wt.% during the first step, significantly surpassing the hydrogen content (1.78 wt.%) associated with aluminum in [HMTA–AlH_3_]_n_. This discrepancy suggests that HMTA initiates decomposition concurrently with dehydrogenation. In [HMTA–AlH_3_]_n_, the decomposition endothermic peak temperature is notably 230 °C lower than that observed for HMTA (266 °C, Figure S2a). This discrepancy could be attributed to the existence of aluminum, which might facilitate the decomposition of HMTA. Because the dehydrogenation process overlaps with the decomposition of HMTA, it is difficult to conclude whether the dehydrogenation process is exothermic or endothermic. However, the overall process during the first step of [HMTA–AlH_3_]_n_ decomposition is exothermic, which is different from the endothermic dehydrogenation of AlH_3_. This difference may arise from the coordination of aluminum atoms by the nitrogen atoms on HMTA. Upon complete decomposition, [HMTA–AlH_3_]_n_ exhibits a mass loss of 76.5 wt.%, with no sublimed HMTA detected by MS. Theoretical considerations, suggest that after [HMTA–AlH_3_]_n_ decomposes into Al, H_2_, other volatile products, and minor solid residues (0.43 wt.% according to the decomposition TGA curve of HMTA), the expected weight loss is approximately 83.57 wt.%. Deviations from this theoretical value, and a higher residue amount (23.50 wt.%), may be ascribed to impurities introduced during the process, such as trace oxidation occurring during sample transfer from the glovebox to the DSC‐TGA apparatus.

The onset decomposition temperature of [TATD–AlH_3_]_n_ is approximately 112 °C, reaching its first peak temperature at 159 °C. Notably, the observed weight loss from the initial exothermic event is approximately 1.36 wt.%, which is lower than the theoretical hydrogen content (H in AlH_3_) of [TATD–AlH_3_]_n_ (1.53 wt.%). This discrepancy might suggest that the decomposition of TATD starts before the dehydrogenation of the AlH_3_ moiety in [TATD–AlH_3_]_n_ is complete, leading to overlapping signals and hampering detailed investigation. Notably, the onset dehydrogenation temperature of [TATD‐AlH_3_]_n_ is lower than that of [HMTA–AlH_3_]_n_, attributed to the elongated Al─H bond lengths in [TATD‐AlH_3_]_n_ (*d*_Al_─_H_ = 1.558 – 1.692 Å) compared to [HMTA–AlH_3_]_n_ (*d*_Al_─_H_ = 1.515 – 1.520 Å). Interestingly, after dehydrogenation of the AlH_3_ moiety on [TATD‐AlH_3_]_n_, decomposition is followed by two steps of weight loss in the TGA curve, which is different from the decomposition pathway of TATD (Figure S2b). It indicates that the presence of aluminum‐nitrogen bonding significantly changed the decomposition pathway of the residue TATD. Upon 430 °C, [TATD–AlH_3_]_n_ shows a mass loss of 56.60 wt.% without vaporized TATD detected by MS, but with another significant H_2_ signal around 293 °C, indicating that the decomposition products from TATD contain H_2_. The theoretical weight loss of [TATD‐AlH_3_]_n_ is 86.34 wt.%, considering Al, H_2_, and other volatile products as decomposition products. The substantial difference between the observed and theoretical values suggests that the decomposition residue likely includes more than metallic aluminum, and there could be aluminum carbides or nitrides.

### 2.2 Hypergolic Properties and Heat of Combustion

Hypergolic properties are crucial for spacecraft and launcher propellant systems.^[^ ^42^, ^43^, ^44^ ^]^ It usually refers to the ability of a propellant combination to ignite spontaneously upon contact with another component, typically an oxidizer or another part of the fuel. In hypergolic systems, the ignition and combustion of the fuel occur without the need for an external ignition source, such as a spark or flame. Hypergolic propellants are commonly used in rocket engines and propulsion systems because of their reliability and simplicity.^[^ ^45^ ^]^ In these systems, a hypergolic fuel and oxidizer are stored separately on the spacecraft or rocket. When the need for propulsion arises, the two components are mixed or brought into contact, initiating a chemical reaction that results in combustion and thrust generation.^[^ ^46^ ^]^ This property is advantageous when rapid and precise ignition is crucial, such as in space missions where ignition reliability is paramount. It eliminates the need for complex ignition systems and reduces the risk of ignition failure during critical maneuvers.

The hypergolic properties of Quin_2_AlH_3_, [TEDA–AlH_3_]_n_, [HMTA–AlH_3_]_n_, and [TATD–AlH_3_]_n_ are evaluated by measuring the ignition delay (ID, i.e., the time between the first contact of the fuel with the oxidizer and ignition) using a typical drop test, in which one 10‐µL drop of white fuming nitric acid (WFNA, 98 % HNO_3_) was released from a fixed height of ∼ 4.5 cm via a 500‐µL Hamilton syringe, into a 4.5‐cm‐high cut pipette fixed on a vial. Each drop test was done thrice and recorded using a Samsung SM‐S901B camera with super slow mode operating at 960 frames s^−1^.^[^ ^47^, ^48^ ^]^

**Figure 4:**
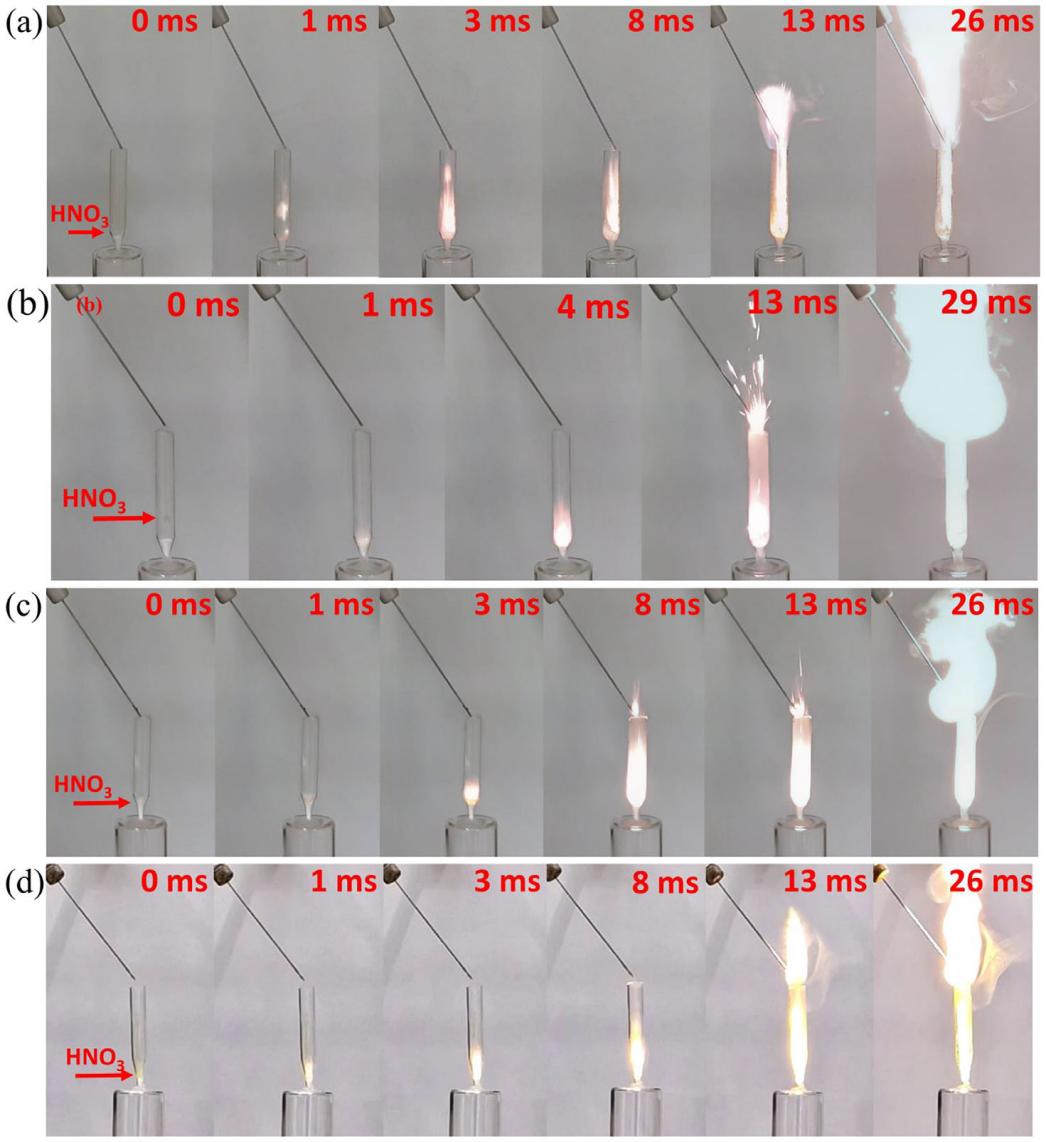
Selected images of hypergolicity drop tests a) Quin_2_AlH_3_, b) [TEDA–AlH_3_]_n_, c) [HMTA–AlH_3_]_n_, d) [TATD–AlH_3_]_n_.

Drop tests with WFNA (Figure 4 and Table 2) revealed rapid ignition for all four explored compounds, with an ultrashort ID of less than 2 ms and intense vigorous flames of more than 10 cm height, lasting 200 ∼ 350 ms. For comparison, a target ID in a functional hypergolic propellant is 50 ms or below.^[^ ^42^ ^]^ Particularly notable is the comparison of the hypergolic activity of Quin_2_AlH_3_, which exhibits aggressive sparks at 1 ms, to the rest of the polymeric compounds with stable ignition processes. These results show that the polymeric structure can improve the stability of the energetic materials explored herein,^[^ ^39^ ^]^ which conforms with the thermal analysis results. [TEDA–AlH_3_]_n_, [HMTA–AlH_3_]_n_, and [TATD–AlH_3_]_n_ show good hypergolic performance, previously not reported in the literature. The flame duration of polymeric compounds decreases in the order of [TEDA–AlH_3_]_n_ > [HMTA–AlH_3_]_n_ > [TATD–AlH_3_]_n_ (Table 2). In particular, [TATD–AlH_3_]_n_ has a slightly higher flame without residue after reacting with the oxidizer.

**Table 2:** Hypergolic properties of the herein explored amine‐AlH_3_ adducts in a drop test using WFNA as an oxidizer.

Compounds	ID [ms]	Flame Duration [ms]	Flame Height [cm]	Flame Color	Observations
Quin_2_AlH_3_	< 1	> 320	> 12	Red	Black residues
[TEDA–AlH_3_]_n_	∼ 1	> 350	> 10	Red – white	Black residues
[HMTA–AlH_3_]_n_	1 ∼ 2	> 340	> 9	Red – white	Black residues
[TATD–AlH_3_]_n_	< 1	> 230	> 10	Yellow – red	No black residue

The heat of combustion, often referred to as the enthalpy of combustion, serves as a crucial metric in evaluating the energy released during the combustion process of fuels.^[^ ^49^, ^50^, ^51^, ^52^ ^]^ This fundamental parameter not only influences the efficiency and performance of propulsion systems but also plays a pivotal role in the design and optimization of rockets, jets, and other propulsion technologies.^[^ ^53^ ^]^ As propellants undergo combustion, releasing heat is not merely a byproduct but a controlled and essential reaction that propels vehicles into motion.^[^ ^54^ ^]^

The experimental constant‐volume combustion heat ($\Delta U_{g}$[kJ g^−1^]& $\Delta U_{c}$[kJ mol^−1^]) values of the herein explored compounds were measured by oxygen bomb calorimetry (refers to higher heating value). The results are shown in Table 3. The combustion reaction of [HMTA–AlH_3_]_n_ is given as an example in Equation (1) as follows:

(1) $$\begin{array}{ccc} & & N_{4}C_{6}H_{12}\cdot \mathrm{Al}H_{3}\left(s\right)+\frac{21}{2}O_{2}\left(g\right)\to 6CO_{2}\left(g\right)+\frac{15}{2}H_{2}O\left(l\right)\\ & & +\;\frac{1}{2}Al_{2}O_{3}\left(s\right)+2N_{2}\left(g\right) \end{array}$$

**Table 3:** Calculated enthalpy of combustion ($\Delta H_{g}$) and experimental combustion heat ($\Delta U_{c}\&\Delta U_{g}$).

Compound	Empirical Formula	Molar Mass [g mol^−1^]	$\Delta H_{g}$ (§) [kJ g^−1^]	$\Delta U_{c}$ [kJ mol^−1^]	$\Delta U_{g}$ [kJ g^−1^]
Aluminum^[^ ^57^ ^]^	Al	27.0	31.1	837.0	31
AlH_3_^[^ ^18^ ^]^	AlH_3_	30.0	41.7	–	–
quinuclidine	C_7_H_13_N	111.2	41.0	4460.2	40.11
TEDA	C_6_H_12_N_2_	112.2	36.6	4094.2	36.49
HMTA	C_6_H_12_N_4_	140.2	30.0	4171.0	29.75
TATD	C_8_H_16_N_4_	168.0	32.4	5495.3	32.71
Quin_2_AlH_3_	C_14_H_29_N_2_Al	252.4	–	10 934.0	43.32
TEDA‐AlH_3_	C_6_H_15_N_2_Al	142.2	–	4874.6	34.28
HMTA‐AlH_3_	C_6_H_15_N_4_Al	170.2	–	5065.2	29.76
TATD‐AlH_3_	C_8_H_19_N_4_Al	198.0	–	6452.8	32.59

(§: Values are calculated according to Equation S1. For clarity, the herein explored compounds were written in monomer form.)

The combustion reaction with O_2_ gas produces solid Al_2_O_3_ along with CO_2_, N_2_ gas, and liquid water. In principle, the enthalpy of combustion ($\Delta H_{c}$) follows the relation: $\Delta \;H_{c}=\Delta U_{c}+\Delta nRT$, where ∆*n* is the change in the total molar quantities of gases during the reaction process, *R* = 8.314 J mol^−1^ K^−1^, and *T* = 298.15 K.^[^ ^55^ ^]^ We have calculated $\Delta H_{g}$ from standard data^[^ ^56^ ^]^ and compared it to $\;\Delta U_{g}$, showing that $\Delta H_{g}$ and $\Delta U_{g}$ are close. Therefore, $\Delta U_{g}$ is directly used for further comparison.

The measured combustion heat value we got from our compounds is significantly higher than commonly used fuels such as hydrazine(19.5 kJ g^−1^) and explosives such as trinitrotoluene (14.9 kJ g^−1^).^[^ ^43^ ^]^ $\Delta U_{c}$ from amines (quinuclidine, TEDA, HMTA, and TATD) decreases in the order of TATD > quinuclidine > HMTA > TEDA (Table 3). Conversely, Quin_2_AlH_3_ has the highest combustion heat and most vigorous flame since each AlH_3_ coordinates with two quinuclidine molecules, which empowers Quin_2_AlH_3_. $\Delta U_{c}$ from [amine‐AlH_3_]_n_ increases in the order of [TEDA–AlH_3_]_n_ < [HMTA–AlH_3_]_n_ < [TATD–AlH_3_]_n_, that is in the same order that $\Delta U_{c}$ of amines increases. Combining with hypergolic properties, it could be concluded that [HMTA–AlH_3_]_n_ and [TATD–AlH_3_]_n_ are the most promising materials for the solid fuels of propellant systems.

### 2.3 Mechanochemical Synthesis

A sustainable synthesis method is one of the most critical factors for developing new propellant candidates.^[^ ^58^ ^]^ Mechanochemistry, that is chemical synthesis enabled or sustained by mechanical force, has evolved over the past few decades into a broadly used and powerful tool for the green synthesis of solid materials.^[^ ^59^, ^60^, ^61^, ^62^ ^]^ As mentioned before, unlike the conventional way to synthesize energetic materials, where a considerable amount of solvents is required during the synthesis process, the mechanochemical method can synthesize solid materials without using a solvent or with only a trace amount of solvents as PCA (process control agent).^[^ ^47^, ^63^ ^]^ Titi et al. have successfully used the mechanochemical method, that is ball milling, to synthesize hypergolic zeolitic imidazolate frameworks (ZIFs).^[^ ^44^ ^]^ Bodach et al. synthesized organometallic aluminum hydrides *R*_2_AlH (*R* = mesityl, *neo*‐pentyl) using a vibratory mill.^[^ ^64^ ^]^ Ortmeyer et al. successfully synthesized [TEDA–AlH_3_]_n_ by ball‐milling TEDA and metallic aluminum powder under hydrogen pressure.^[^ ^39^ ^]^ In the present work, we further investigate the mechanochemical syntheses of Quin_2_AlH_3_, [HMTA–AlH_3_]_n_, and [TATD–AlH_3_]_n_.

**Figure 5:**
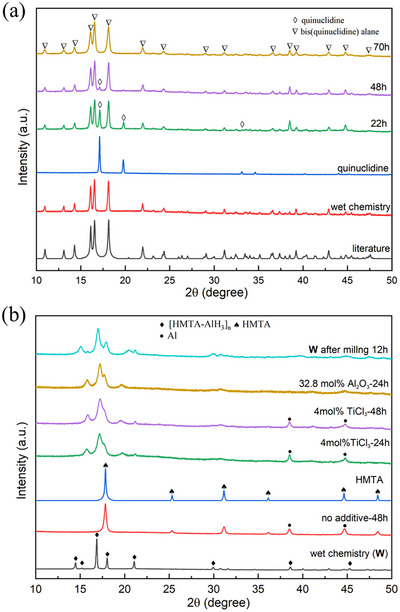
PXRD patterns of the samples resulting from different ball milling conditions (a) quinuclidine and aluminum after 22, 48, and 70 hours of milling at 500 rpm under 180 bar hydrogen pressure, as specified; (b) HMTA and aluminum with and without additives (TiCl_3_, Al_2_O_3_) after 24 hours and 48 hours of milling at 500 rpm under 180 bar hydrogen pressure, as specified.

In the case of Quin_2_AlH_3_, when milling Quin and Al in the presence of H_2_ is carried out at a frequency of 500 rpm on a planetary milling machine, the formation of Quin_2_AlH_3_ is stretched over about 22 hours (Figure 5a). The most prominent reflections of aluminum at 38.5° and 44.8° (2*θ*, CuKα_1_) overlap with the reflections from Quin_2_AlH_3_. Therefore, the reflections of quinuclidine are used to indicate the complete extent of the reaction. The characteristic reflections of quinuclidine at 17.1° and 19.7° (2*θ*) almost vanish after milling for 48 hours, reaching complete conversion at 70 hours to Quin_2_AlH_3_. The ^1^H NMR spectra of samples obtained after 70 hours of milling are compared to those of the product from wet chemistry, as shown in Figure S3, confirming the complete conversion of quinuclidine. Thermal analysis of the 70 hours ball milling product (Figure S4a) reveals the endothermic decomposition in two steps, accompanied by hydrogen release and sublimation of quinuclidine, similar to the wet chemistry method product. However, the onset dehydrogenation temperature of the milled product is around 52 °C, and therewith 23 °C lower than that of the sample synthesized by the wet chemistry method, indicating that synthesis methods do not notably affect thermal behaviors but could lead to lower thermal stability. This could be due to reduced particle sizes or traces of metal impurities from the abrasion of the milling jar and balls.

[HMTA–AlH_3_]_n_ was also synthesized by ball milling with additives (TiCl_3_ and Al_2_O_3_) that could act as catalysts or/and PCAs. Interestingly, without additives, milling could not lead to any formation of products, and HMTA and aluminum were still clearly detected after 48 hours of milling in the PXRD patterns of milled powders (Figure 5b). In contrast, when additives (TiCl_3_ and Al_2_O_3_) were employed, no HMTA was detected after 24 hours of milling, and the new set of reflections for the desired [HMTA–AlH_3_]_n_ proved a successful synthesis. However, the reflections of the product were rather broad, indicating a certain extent of disorder in the polymeric structure of [HMTA–AlH_3_]_n,_ and a small amount of unreacted aluminum was also detected. Therefore, we extended the milling time to 48 hours, expecting better conversion, but the product is similar to that from the 24 hours milling time. Al_2_O_3_ was employed in the milling process to investigate the effects of additives further. The product has a similar pattern to the product from milling with TiCl_3_. This shows that both TiCl_3_ and Al_2_O_3_ can have a similar positive effect on the synthesis process during ball milling. In the case of TiCl_3_, according to the previous report,^[^ ^65^ ^]^ it can act as an electron source to facilitate the hydrogenation of aluminum element and further stabilize the formed Al─H species and promote the adducts formation.^[^ ^66^ ^]^

In case of Al_2_O_3_ the experimental effect is obvious while its nature can result from various scenarios, such as modification of the impact or its role as strong Lewis acidic support for the reactants or traces of metals from the grinding media.

A similar PXRD pattern was observed in sample **W** after 12 hours of ball milling, which further confirmed that the product from the ball milling process is the desired product with disordered packing because of mechanical force. Additionally, we explored the effects of different ball milling speeds (Figure S5). At 400 rpm, a decrease in the conversion rate was observed, indicated by the increased intensity of aluminum diffraction compared to the product at 500 rpm, while at 600 rpm, HMTA and metallic aluminum reappeared, suggesting product decomposition due to excessive milling speed. Based on the comparison of PXRD patterns from ball milling products, the product obtained from milling with TiCl_3_ for 48 hours represents the most optimal product obtained through ball milling.

Thermal analysis of the product obtained from milling with TiCl_3_ for 48 hours reveals exothermic decomposition in two steps, starting at around 128 °C, and one endothermic decomposition step at a peak temperature of 263 °C (Figure S4b). Compared to the decomposition process of the sample from the wet chemistry method (Figure 3b), the ball milling sample has a lower onset dehydrogenation temperature and two exothermic peaks at 162 °C and 180 °C, respectively. It indicates the lower thermal stability of the ball‐milled product and different hydrogen release pathways at 162 °C that could be attributed to the disorder of the polymeric structure.

The synthesis of [TATD─AlH_3_]_n_ by ball milling has been investigated with and without the additives (TiCl_3_ and Al_2_O_3_) at 500 rpm for 24 hours under hydrogen pressure. Unfortunately, no conversion was observed (Figure S6). This could be due to the relatively low stability of [TATD─AlH_3_]_n_, which starts to decompose at 112 °C and release heat, so that, during ball milling, the formed [TATD─AlH_3_]_n_ decomposes directly.

## 3 Conclusion

We demonstrated for the first time that amine‐AlH_3_ adducts have an excellent hypergolic response (ID < 2 ms) and combustion performance ($\Delta U_{g}$ > 29 kJ g^−1^), which are comparable to reported energetic materials and make them potential candidates for a new generation of green solid fuels. DSC–TGA–MS has studied the thermal stability of the samples, and the results indicate that amine–AlH_3_ adducts with polymeric structure ([TEDA–AlH_3_]_n_, [HMTA–AlH_3_]_n_, and [TATD–AlH_3_]_n_) are more stable than that with monomer crystal structure (Quin_2_AlH_3_). An environmentally friendly and convenient method, the mechanochemical process, successfully synthesizes Quin_2_AlH_3_ and [HMTA–AlH_3_]_n_, besides [TEDA–AlH_3_]_n_^[^ ^20^ ^]^ reported previously. The successful mechanical synthesis of Quin_2_AlH_3_, [HMTA–AlH_3_]_n,_ and [TEDA–AlH_3_]_n_ also demonstrates their high mechanical stability against impact and frictional forces. The adducts reported in this study contain only aluminum, carbon, nitrogen, and hydrogen in their structures. Thus, both the educts and the products H_2_O, CO_2_, N_2_, and Al_2_O_3_ formed during combustion are free of environmentally hazardous heavy metals or other problematic compounds. This work offers new perspectives for a possible new generation of solid fuels and inspires a new method of synthesizing energetic materials.

## Supporting Information

The authors have cited additional references within the Supporting Information.^[67–74]^

## Conflict of Interest

The authors declare no conflict of interest.

## Supporting information



Supporting Information

Supporting Information

Supporting Information

Supporting Information

Supplemental Movie 1

Supplemental Movie 2

Supplemental Movie 3

Supplemental Movie 4

## Data Availability

The data that support the findings of this study are available from the corresponding author upon reasonable request.
