# Supplementary material for: Amine‐AlH3 Adducts as Energetic Materials for a New Generation of Solid Fuels
Source: Chemistry. 2025 Jun 26;31(40):e202501163. doi: 10.1002/chem.202501163 (PMC12271996; doi:10.1002/chem.202501163)
Supplement: Supplementary file 1 — Supporting Information [file CHEM-31-e202501163-s002.docx]

Supporting Information

Amine-AlH_3_ Adducts as Energetic Materials for a New Generation of Solid Fuels

Xiaoran Liu^[a]^, Jochen Ortmeyer^[a]^, Alexander Bodach^[a]^, Hilke Petersen^[a]^, and Michael Felderhoff*^[a]^

[a] Max-Planck-Institut für Kohlenforschung, Kaiser-Wilhelm-Platz 1, Mülheim an der Ruhr, 45470, Germany

E-mail: felderhoff@kofo.mpg.de

**Experimental methods**

All operations were carried out in an anaerobic and anhydrous argon atmosphere by working with a Schlenk line or in a glovebox with a circulation purifier operating at <1 ppm of O_2_ and H_2_O, respectively. Aluminum chloride (AlCl_3_, 99.99 %, Sigma-Aldrich), aluminum powder (99 %, Alfa Aesar), and hexamethylenetetramine (HMTA, 99.99 %, Sigma-Aldrich) were used as received. Lithium alanate (LiAlH_4_, 95 %, Sigma-Aldrich) was purified via recrystallization from diethyl ether, and tetraazatricyclododecane (TATD) was synthesized from paraformaldehyde and ethylenediamine according to the literature.^[67]^ Its X-ray structure is shown in **Figure S7** (CCDC 2386676). Titanium chloride (TiCl_3_) was synthesized as described in the literature. ^[68]^ Diethyl ether (Et_2_O) was dried (< 5 ppm H_2_O) before use with a standard procedure.^[69]^

**Safety Note**:

Aluminum hydride compounds should generally be handled with care under inert gas conditions. In the presence of moisture, they decompose and release hydrogen gas, which can form an explosive gas mixture with air.

The synthesized amine-AlH_3_ adducts are more stable compared to pure AlH_3_, but also decompose slowly in the presence of air and moisture, so these compounds should also be handled under inert gas conditions.

Since some of the described materials could be synthesized during ball milling in a hydrogen gas atmosphere, these materials seem to have a high resistance to impact and shock forces, and no decomposition due to overheating during the milling process could be observed.

**Amines used in this Project**


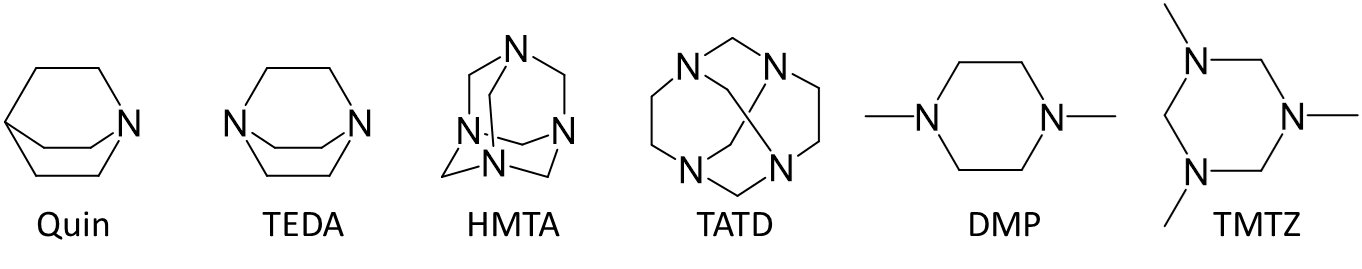


Explored tertiary amines in this Project

In the above-mentioned explored tertiary amines, it is difficult to get pure and stable adducts from DMP (N, N dimethylpiperazine) and TMTZ (1, 3, 5-trimethyl-1, 3, 5-triazinane).

| Amines | N content (wt.%) | Coordination capability | Basicity (pKa) |
| --- | --- | --- | --- |
| Quin | 12.6 | 1 | 11.3 (strong) |
| TEDA | 25.0 | 2 | pKa 1 = 3.0; pKa 2 = 8.7 (strong) |
| HMTA | 40.0 | 4 | 4.9 (weak) |
| TATD | 33.3 | 4 | 8.5 - 9.5 (medium) |
| DMP | 24.6 | 2 | pKa 1 = 9.8; pKa 2 = 5.7 (strong) |
| TMTZ | 32.6 | 3 | 8.5 - 9.5 (medium) |

**NMR Spectroscopy**

Solution ^1^H NMR spectra (300 MHz, 25 °C ) were recorded on Bruker AV 300nano in 5 mm diameter NMR tubes. The chemical shifts for ^1^H were given in ppm relative to the residual solvent (C_6_D_6_, *δ* = 7.16 ppm). NMR data is reported as follows: chemical shift δ in ppm; multiplicity (hept = heptet, m = multiplet, bs = broad singlet; coupling constants in Hz).

**Infrared Spectroscopy**

Transmission FTIR spectra were collected using a Nicolet Magna-IR 560 spectrometer with a mercury–cadmium–telluride (MCT) detector. Measurements were carried out in the 4000 ~ 400 cm^–1^ range with a 4 cm^–1^ resolution (60 scans per spectrum). The 2 ~ 3 mg samples were pressed into pellets mixed with ~200 mg KBr with a mortar inside the glovebox and pelletized. IR data is reported with abbreviations: s = strong; w = weak.

**Powder X-ray Diffraction and Structure Determination**

The PXRD measurements were performed on a STADI P diffractometer (STOE and Cie GmbH, Darmstadt, Germany) in transmission mode (capillary = 0.7 mm) using Cu Kα1 radiation. The instrument had a primary Ge (111) monochromator and a position-sensitive detector (PSD) system filled with 90 % argon with 10 % CH_4_. The diffraction patterns, in general, were recorded with a step size of 0.5° (2*θ*) and a counting time of 45 s step^-1^. The PXRD data used for structure determination is collected with a step size of 0.5° (2*θ*) and a counting time of 80 s step^‑1^. The crystal structures of [HMTA–AlH_3_]_n_ were solved with real space methods via the program DASH ^[70]^, and molecular models of the compounds were derived from the known structures of HMTA ^[71]^ and AlH_3_.^[72]^ The solved structures were refined via the Rietveld method with *DiffracPlus TopasV6* software (Bruker AXS GmbH, Karlsruhe, Germany).^[73]^ The crystal structures of [TATD–AlH_3_]_n_ were solved with a simulated annealing approach and refined via the Rietveld method with *DiffracPlus TopasV6* software.^[73]^

TATD (CCDC 2386676) data were from a single structure we synthesized (**Table S1**). The selected crystallographic data of [HMTA–AlH_3_]_n_ and [TATD–AlH_3_]_n_ are shown in **Tables S2 & 3**.

**Table S1** Crystal data and summary of X-ray diffraction data for TATD.

| empirical formula | C_8_ H_16_ N_4_ |  |
| --- | --- | --- |
| molar mass [g mol^-1^] | 168.25 |  |
| Temperature [K] | 100(2) |  |
| Wavelength [Å] | 0.71073 |  |
| Crystal system | Tetragonal |  |
| Space group | *I ¯*4 2*m*, (no. 121) |  |
| *a* [Å] | a = 7.3853(1) |  |
| *b* [Å] | b = 7.3853(1) |  |
| *c* [Å] | c = 7.572(2) |  |
| *α* [°] | 90 |  |
| *β* [°] | 90 |  |
| *γ* [°] | 90 |  |
| Volume [Å^3^] | 412.99(1) |  |
| *Z* | 2 |  |
| Density (calculated) [mg·m^-3^] | 1.353 |  |
| Absorption coefficient [mm^-1^] | 0.087 |  |
| *F*(000) | 184 e |  |
| Crystal size [mm^3^] | 0.1 x 0.1 x 0.09 |  |
| *θ* range for data collection | 3.854 to 33.147° |  |
| Reflections collected | 1777 |  |
| Independent reflections | 430 [Rint = 0.0354] |  |
| Goodness-of-fit on F^2^ | 1.170 |  |
| Final R indices [I>2σ(I)] | R_1_ = 0.0591 | wR^2^ = 0.1675 |
| R indices (all data) | R_1_ = 0.0655 | wR^2^ = 0.1716 |
| Largest diff. peak and hole [Å^-3^] | 0.4 and -0.2 e· |  |

CCDC 2386676 contains the supplementary crystallographic data for [HMTA-AlH_3_]_n_.^[74]^

**Table S2** Selected crystallographic data of [HMTA-AlH_3_]_n_.

| empirical formula | C_6_H_15_AlN_4_ |
| --- | --- |
| molar mass [g mol^-1^] | 169.7 |
| crystal system | orthorhombic |
| space group | *Cmcm*, (no. 63) |
| *a* [Å] | 9.8151(1) |
| *b* [Å] | 7.2085(1) |
| *c* [Å] | 12.2019(1) |
| *α* [°] | 90 |
| *β* [°] | 90 |
| *γ* [°] | 90 |
| Volume [Å^3^] | 863.32(1) |
| *Z, Z’* | 4, 0.25 |
| R_wp_ | 2.74 |
| Al–N bond length [Å] | 2.190(2) |
| Al–H bond length [Å] | 1.515 - 1.520 |

CCDC 2386632 contains the supplementary crystallographic data for [HMTA-AlH_3_]_n_.^[76]^

**Table S3** Selected crystallographic data of [TATD-AlH_3_]_n_.

| empirical formula | C_8_H_19_AlN_4_ |
| --- | --- |
| molar mass [g mol^-1^] | 197.7 |
| crystal system | monoclinic |
| space group | *P*12_1_*/m*1, (no. 11) |
| *a* [Å] | 11.0539(1) |
| *b* [Å] | 7.4449(1) |
| *c* [Å] | 6.1596(1) |
| *α* [°] | 90 |
| *β* [°] | 96.199(1) |
| *γ* [°] | 90 |
| Volume [Å^3^] | 503.94(1) |
| *Z, Z’* | 2, 0.5 |
| R_wp_ | 3.56 |
| Al–N bond length [Å] | 2.291(6) |
| Al–H bond length [Å] | 1.558 - 1.692 |

CCDC 2386633 contains the supplementary crystallographic data for [TATD-AlH_3_]_n_.^[74]^

**DSC‑TGA-MS Measurement**

The measurements were performed on a Mettler Toledo DSC/TGA 1 with a gas controller GC 200 (argon flow: 50 mL min^-1^) combined with a ThermoStar GSD 300T2 Mass Spectrometer gas analyzer. Approximately 10 mg of samples were loaded into an aluminum crucible and heated from 30 to 430 °C (heating rate, 1 or 5 °C min^‑1^) under a protective flow of argon (gas flow rate, 50 mL min^-1^). Mass spectra were collected in the multiple ion detection (MID) mode for the detection of H_2_ (2.00 g mol^-1^) and quinuclidine (110.91 g mol^‑1^).

**Hypergolic Testing**

In performing drop tests, one 10-μL drop of white fuming nitric acid (WFNA) was released from a fixed height of ~ 4.5 cm via a 500-μL Hamilton syringe (Hamilton CO. Reno, Nevada). The pipettes for the tests had a height of 4.5 cm and were fixed on the vials. A Samsung SM-S901B mobile phone camera with super slow mode was used to record the event at 960 frames per second (fps). The ignition delay (ID) was then measured by capturing the time points between the initial contact of the droplet with the sample and the visible first emission of light.

**Bomb Calorimetry**

The bomb calorimetry measurements were carried out on an IKA C 6000 isoperibol calorimeter system (IKA-Werke GmbH & Co. KG). The certified benzoic acid (IKA C723, ~ 1.0 g, two tablets) was adopted by combustion in an oxygen atmosphere at a pressure of 34 bar to calibrate the calorimeter. Approximately 100 mg of the sample was pressed into a tablet and then sealed in the acetobutyrate capsule (IKA C10). The tablet was sufficiently burned in a 34-bar atmosphere of pure oxygen in a combustion crucible (IKA C5) in a C 6010 decomposition vessel.

**Synthetic methods**

***Synthesis of* *Quin_2_AlH_3_***

*Wet Chemistry Method*: A solution of AlCl_3_ (0.670 g, 0.50 mmol ) in THF (10 mL) was added to a solution of LiAlH_4_ (0.570 g, 1.50 mmol) in THF (40 mL). After 10 min stirring at room temperature, the mixture was cooled to – 115 °C (ethanol/liquid nitrogen) and stirred for 10 min. LiCl was removed by filtration, and then the filtrate was added to a cooled (– 115 °C, ethanol/liquid nitrogen) solution of quinuclidine (0.445 g, 4.00 mmol) in THF (80 mL). This mixture was allowed to heat slowly to room temperature while stirring overnight. The solvent was removed in vacuo for 4 - 5 h, and then the product was collected as a colorless solid with a yield of 61 % (0.309 g, 1.95 mmol).

*Mechanochemical Synthesis*: Quin_2_AlH_3_ was obtained mechanochemically using Fritsch Pulverisette 6 planetary mono mill (**Figure S8**) by ball-milling metallic aluminum powder (0.057 g, 2.10 mmol) and quinuclidine (0.467 g, 4.20 mmol) in a 50-mL stainless steel milling jar, using three stainless steel milling balls 15 mm in diameter (mass: 3*13.6 g) and five 10 mm in diameter (mass: 5*4.0 g). The mixture was milled for varying times (22, 48, and 70 h) at 500 rpm under 180 bar hydrogen pressure and taken for PXRD analysis (hydrogen was recharged to 180 bar after every sample collection). The milling program implied the repetition of the same two steps cyclically – first, 30 min of milling and then a 10 min break – until the target milling time was finally reached. Rotation was inverted when moving from one repetition to the other to improve the homogeneity of the treatment.

^1^H NMR of Quin_2_AlH_3_ (300 MHz, C_6_D_6_, 25 °C) *δ* (ppm) = 3.57 (bs, 3H, Al-H), 3.11 – 2.82 (m, 12H, N-CH_2_), 1.34 (hept, *J* = 3.2, 2H, (CH_2_)_2_C**H**), 1.24 – 0.98 (m, 12H, CH_2_-C**H**_2_-CH).

^13^C NMR (75 MHz, C_6_D_6_, 25 °C) *δ* (ppm) = 46.73, 26.04, 21.97.

IR (KBr pellets): ϑ_max_ (cm^-1^) 2950 (m), 2883 (w), 2869 (m), 1682 (m, Al-H), 1459 (w), 1409 (w), 1317 (w), 1278 (w), 1203 (w), 1114 (w), 1053 (w), 989(w), 889 (m, AlH_3_ out of plane bend), 829 (w), 761 (s, AlH_3_ in-plane bend), 704 (s, N-Al-H bend).

***Synthesis of [HMTA–AlH_3_]_n_***

*Wet Chemistry Method*: A solution of AlCl_3_ (0.267 g, 2.00 mmol) in Et_2_O (20 mL) was added to a solution of LiAlH_4_ (0.227 g, 6.00 mmol) in Et_2_O (100 mL). After 10 min stirring at room temperature, the mixture was cooled to – 78 °C (acetone/dry ice) and stirred for 10 min. LiCl was removed by filtration, and then the filtrate was added to a cooled (– 78 °C, acetone/dry ice) solution of HMTA (1.121 g, 8.00 mmol) in Et_2_O (100 mL). This mixture was allowed to heat slowly to room temperature while stirring overnight. The product was isolated by filtration, washed with Et_2_O, and dried under vacuum for 4 - 5 h. Yield: 91 % (1.235 g, 7.28 mmol) as a colorless solid.

*Mechanochemical Synthesis*: [HMTA–AlH_3_]_n_ was obtained mechanochemically using Fritsch Pulverisette 6 planetary mono mill (Figure S6) by ball-milling metallic aluminum powder (0.081 g, 3.00 mmol), HMTA (0.421 g, 3.00 mmol), and TiCl_3_ (0.019 g, 0.12 mmol) or Al_2_O_3_ (0.036 g, 0.35 mmol) as the additives in a 50-mL stainless steel milling jar, using three stainless steel milling balls 15 mm in diameter (mass: 3*13.6 g) and five 10 mm in diameter (mass: 5*4.0 g). The mixture was milled for varying times (22 and 48 h) at 500 rpm under 180 bar hydrogen pressure and taken for PXRD analysis (hydrogen was recharged to 180 bar after every sample collecting). The milling program implied the repetition of the same two steps cyclically – first, 30 min of milling and then a 10 min break – until the target milling time was finally reached. Rotation was inverted when moving from one repetition to the other to improve the homogeneity of the treatment.

IR (KBr pellets): ϑ_max_ (cm^-1^) 2980 (w), 2944 (m), 2924 (w), 2875 (w), 1760 (s, Al-H), 1445 (m), 1373 (w), 1328 (w), 1242 (s), 1228 (s), 1058 (m), 1024 (s), 991 (s), 929 (s), 879 (s, AlH_3_ out of plane bend), 837 (s), 821 (s), 761 (s, AlH_3_ in-plane bend), 698 (s, N-Al-H bend).

***Synthesis of [TATD–AlH_3_]_n_***

*Wet Chemistry Method*: A solution of AlCl_3_ (0.250 g, 1.87 mmol ) in Et_2_O (20 mL) was added to a solution of LiAlH_4_ (0.214 g, 5.64 mmol) in Et_2_O (100 mL). After 10 min stirring at room temperature, the mixture was cooled to – 78 °C (acetone/dry ice) and stirred for 10 min. LiCl was removed by filtration, and then the filtrate was added to a cooled (– 78 °C, acetone/dry ice) solution of TATD (0.420 g, 2.50 mmol) in Et_2_O (100 mL). Different ratios of TATD towards AlH_3_ were also synthesized to investigate other possible products (**Figure S9**). This mixture was allowed to heat slowly to room temperature while stirring overnight. The product was isolated by filtration, washed with Et_2_O, and dried under vacuum for 4 - 5 h. Yield: 90 % (0.445 g, 2.25 mmol) as a colorless solid.

IR (KBr pellets): ϑ_max_ (cm^-1^) 2961 (s), 2914 (s), 2866 (s), 1765 (s, Al-H), 1732 (s, Al-H), 1704 (s, Al-H), 1459 (s), 1398 (m), 1384 (w), 1356 (s), 1303 (s), 1267 (s), 1139 (s), 1100 (s), 1029 (s), 977 (s), 898 (s, AlH_3_ out of plane bend), 794 (s), 760 (s, AlH_3_ in-plane bend).

**Experimental methods**


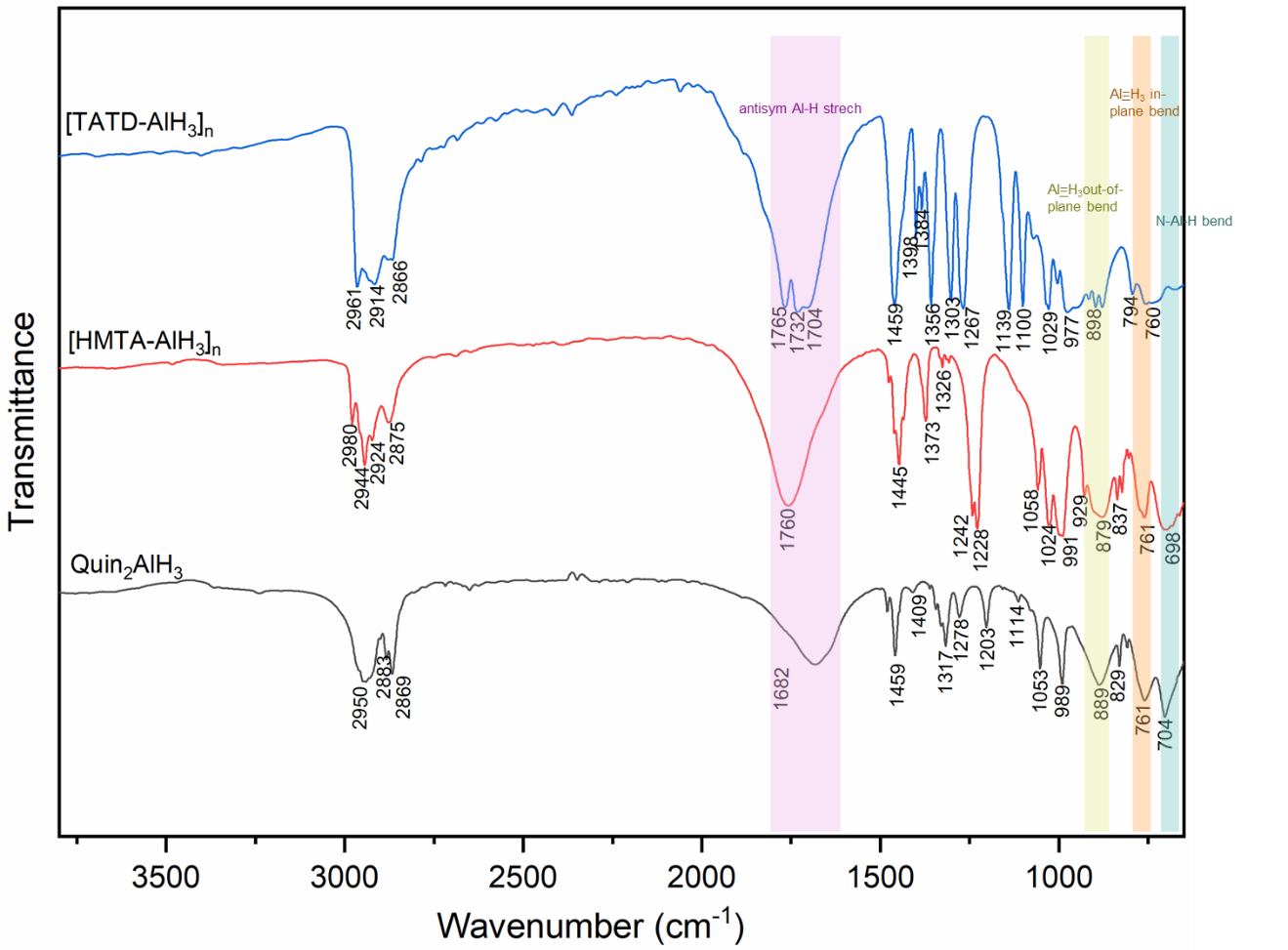


**Figure S1.** FTIR spectra of Quin_2_AlH_3_, [HMTA-AlH_3_]_n_ and [TATD-AlH_3_]_n_.


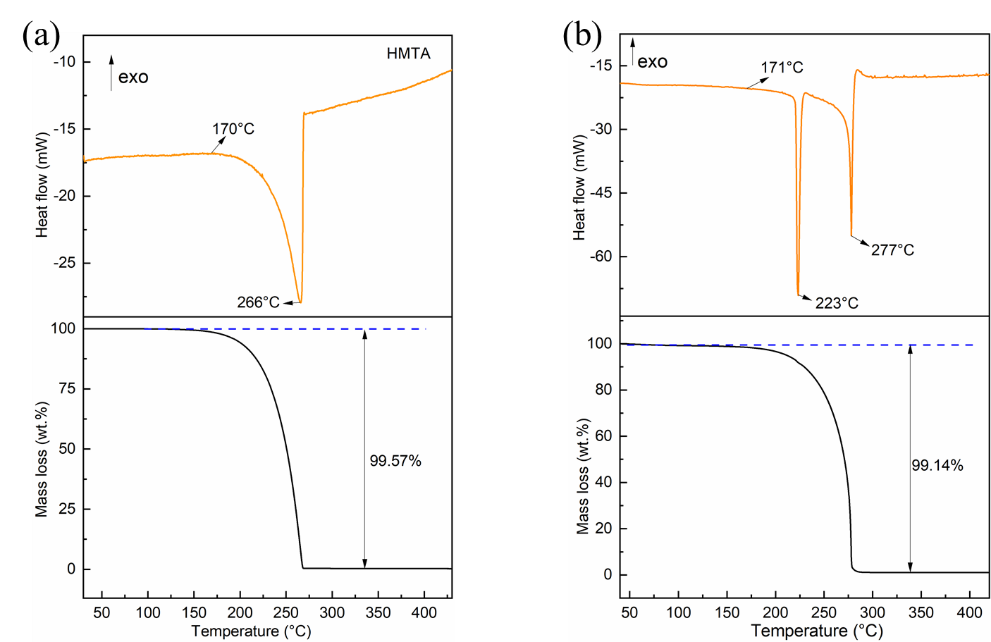


**Figure S2.** DSC (top) and TGA (bottom) curves of (a) HMTA and (b) TATD.


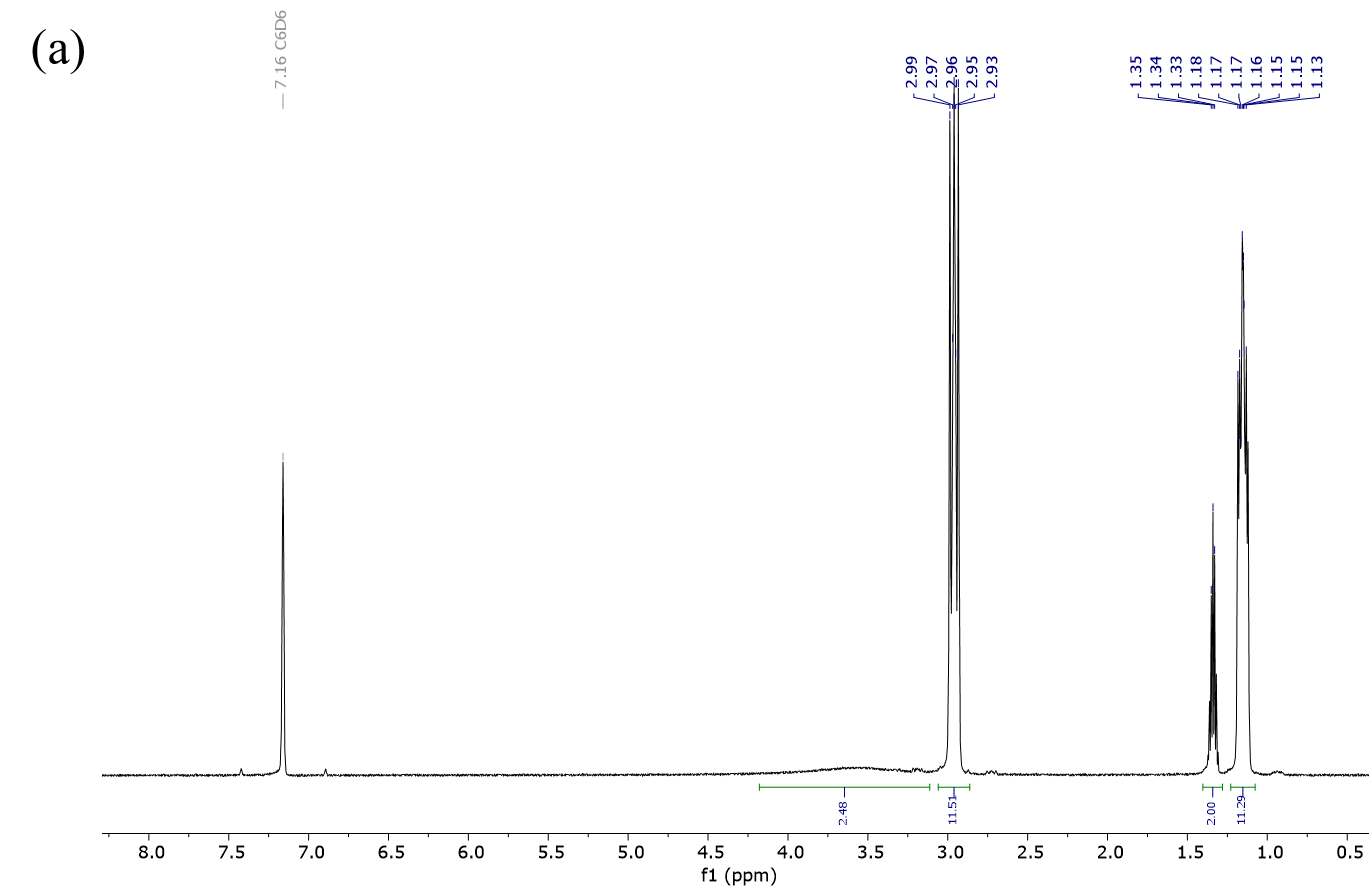


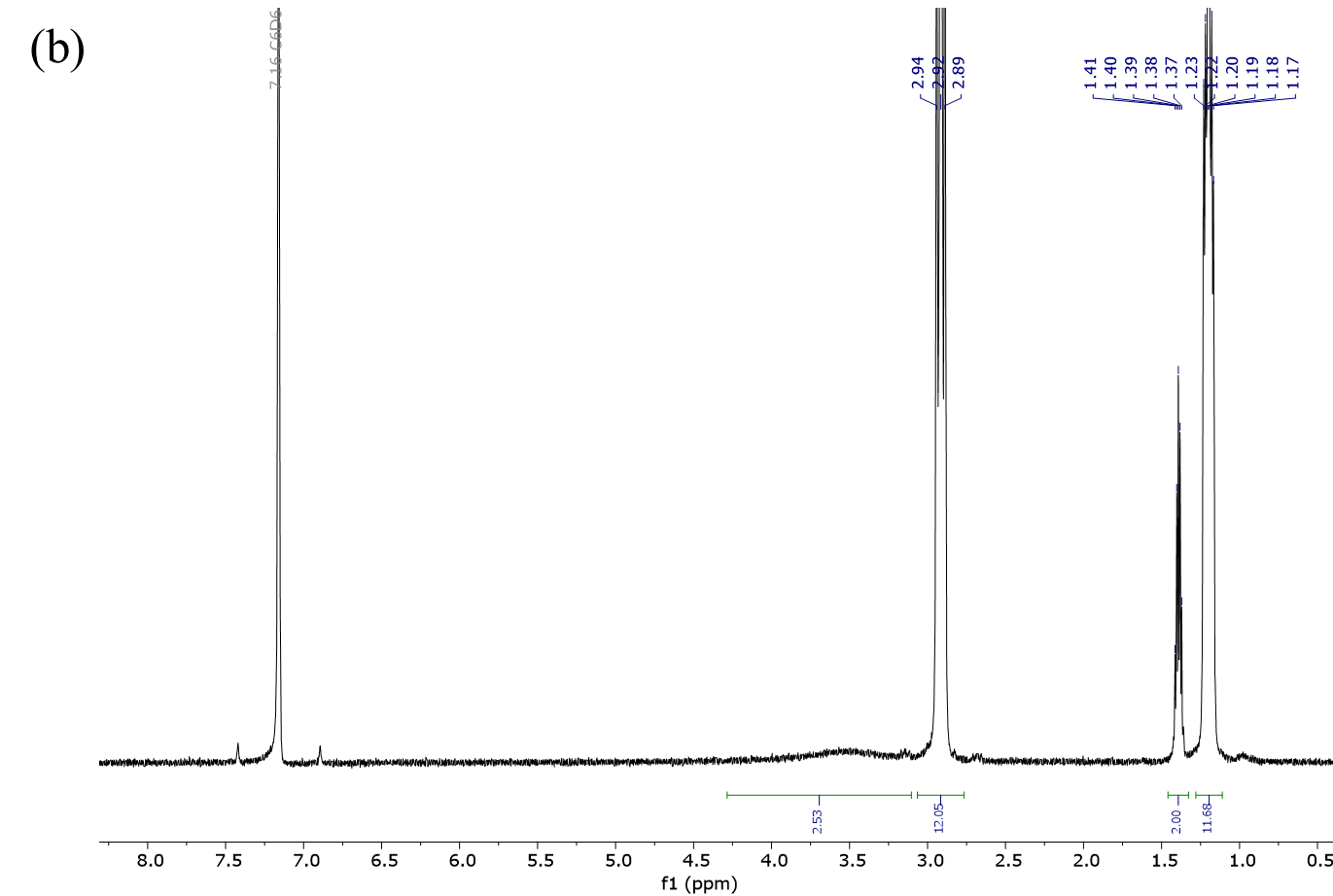


**Figure S3.** NMR spectra (300 MHz, C_6_D_6_, 25 °C) of Quin_2_AlH_3_ from (a) wet chemistry and (b) ball milling.


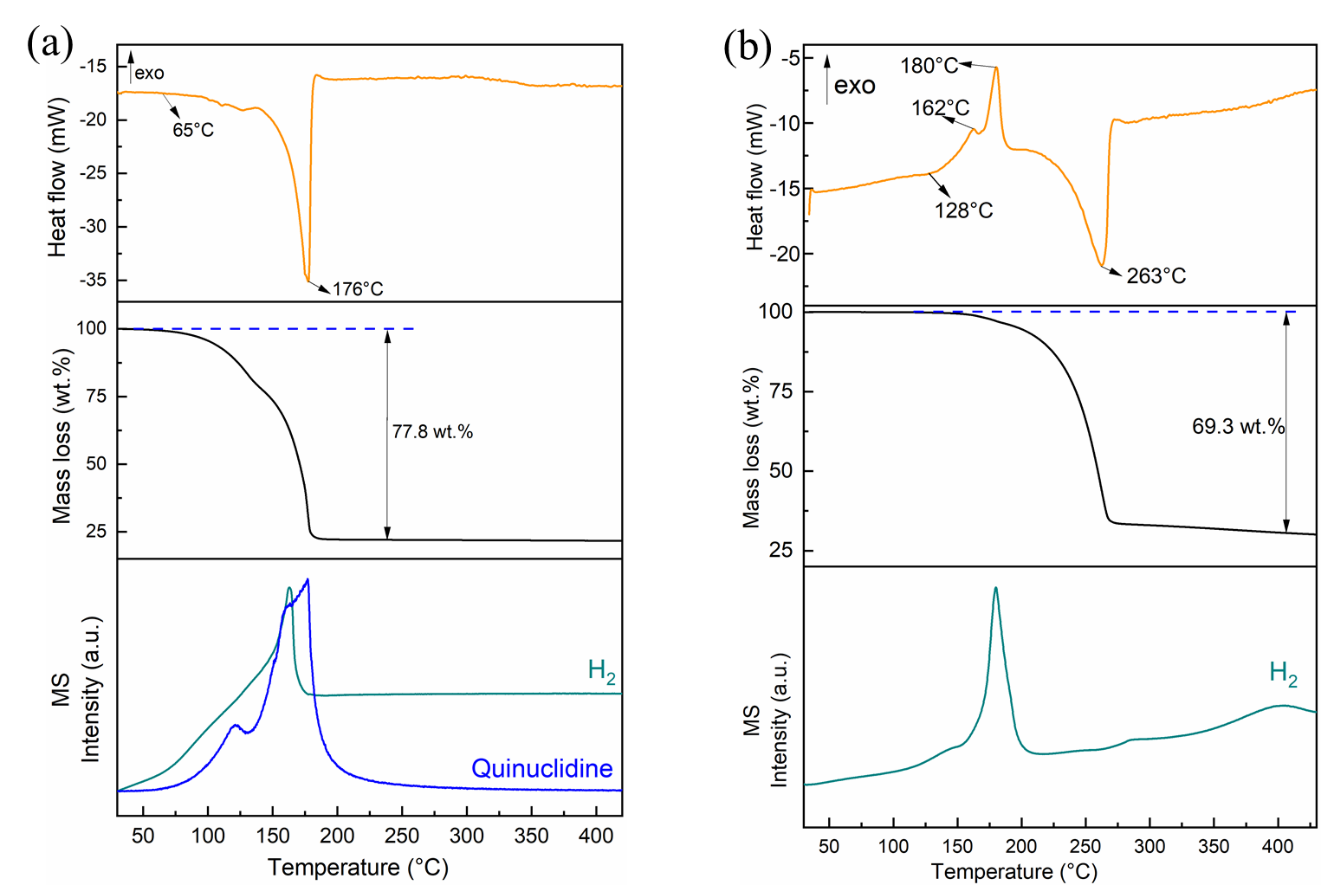


**Figure S4.** DSC (top), TGA (middle), and MS (bottom) curves of the sample from ball milling of (a) Al and quinuclidine under 180 bar H_2_ pressure after 70 h, (b) Al and HMTA under 180 bar H_2_ pressure after 48 h.


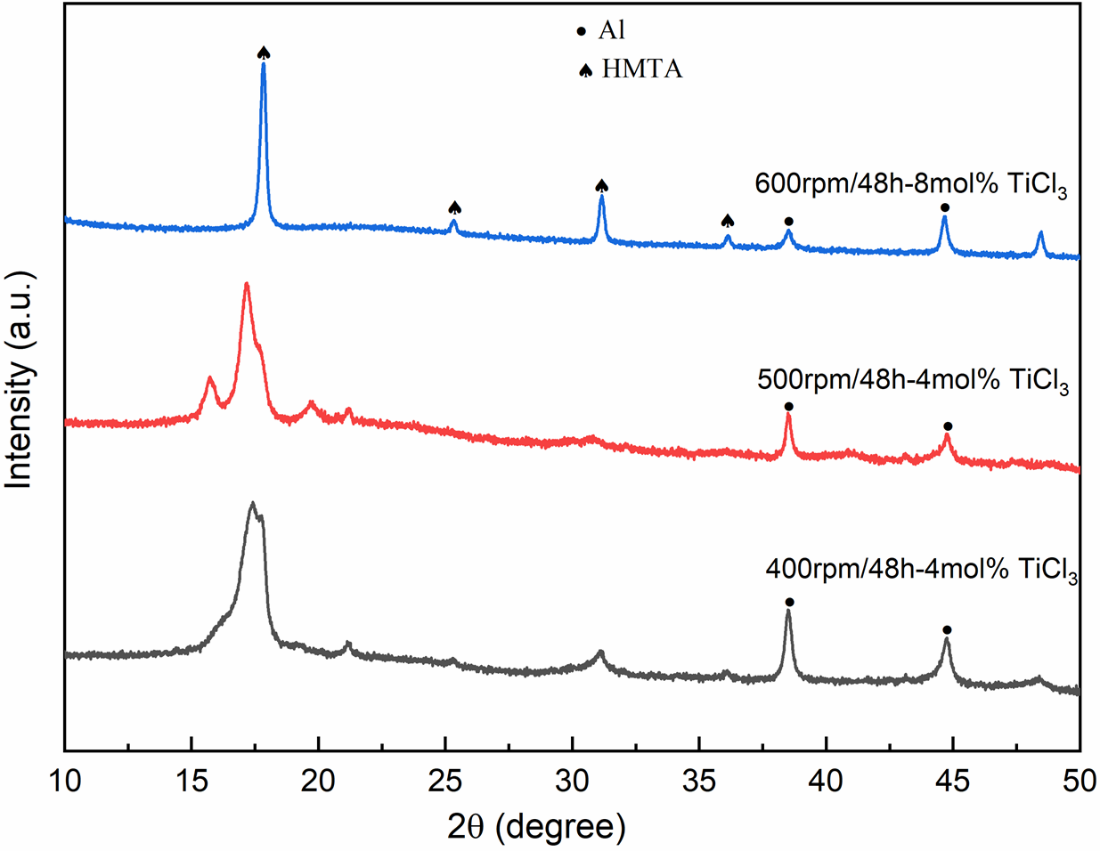


**Figure S5.** PXRD patterns of the samples resulting from HMTA and aluminum after ball milling with different milling speeds under 180 bar hydrogen pressure.





**Figure S6.** PXRD patterns of the sample ([TATD-AlH_3_]_n_) via wet chemistry (W) and samples (TATD + Al) via ball milling with/without the additives (TiCl_3_, Al_2_O_3_) after 24 h of milling at 500 rpm under 180 bar hydrogen pressure, as specified.


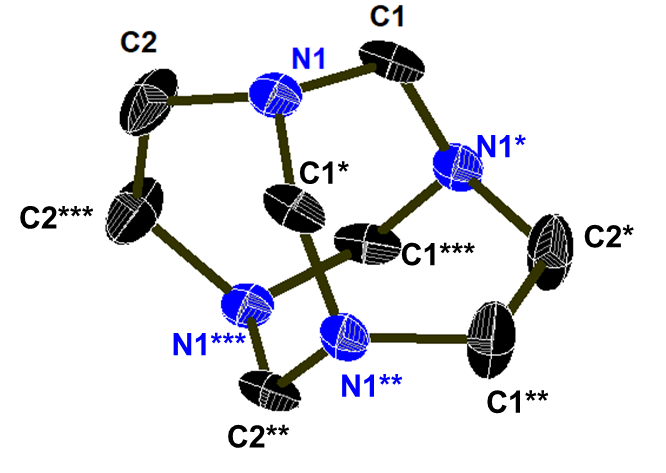


**Figure S7.** Molecular structure and atom-numbering scheme of TATD obtained from our synthesis. Thermal ellipsoids are drawn at the 50 % probability level. H atoms have been omitted for clarity.


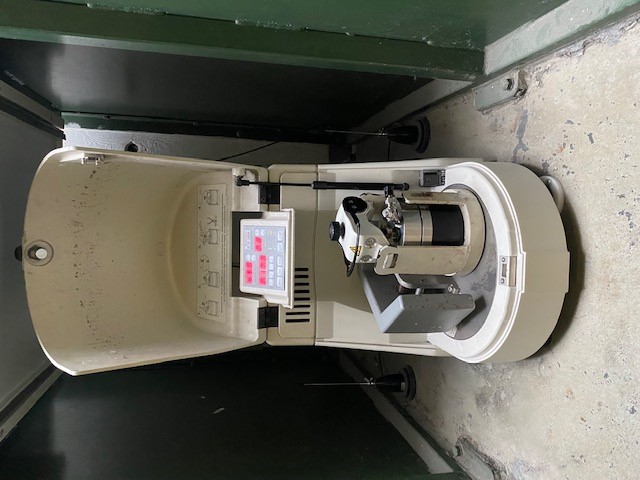


**Figure S8.** Fritsch Pulverisette 6 planetary mono mill.


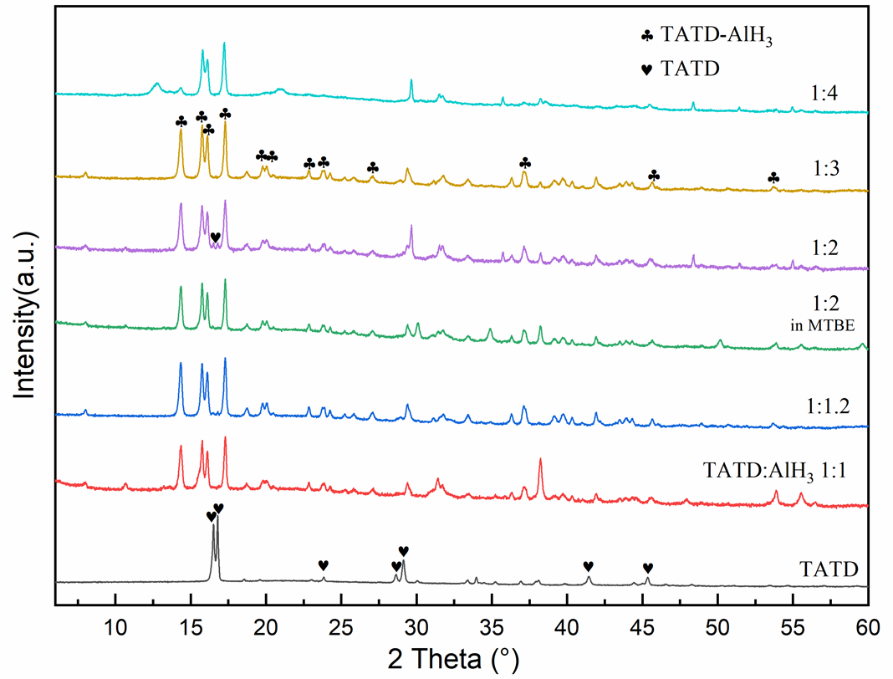


**Figure S9**. PXRD patterns of wet chemistry samples from different ratios of TATD to AlH_3_.

**Equation S1**:

N(CH_2_CH_2_)_3_CH (s) + $\frac{41}{4}$ O_2_ (g) → 7 CO_2_ (g) + $\frac{13}{2}$ H_2_O (*l*) + $\frac{1}{2}$ N_2_ (g)

$$\Delta H=7 \Delta H_{f, CO_{2}}^{0}+ \frac{13}{2} \Delta H_{{f,H}_{2}O}^{0}-\Delta H_{f,N\left( CH_{2}CH_{2} \right)_{3}\mathrm{CH}}^{0}$$

= $7\times\left( -393.5 kJ \mathrm{mol}^{-1} \right)+\frac{13}{2}\times\left( -285.8 kJ {mol}^{-1} \right)-\left( -55.1 kJ {mol}^{-1} \right)$

= $-4557.1 kJ {mol}^{-1}$

$M_{N{({CH}_{2}\mathrm{CH}_{2})}_{3}\mathrm{CH}}=111.2 g {mol}^{-1}$

$$\text{∆}\text{H}_{\text{g}}= - 40.98 kJ g^{-1}$$

Ref. NIST Chemistry WebBook, SRD 69.

**Reference:**

[67] M. B. Peori, K. Vaughan, D. L. Hooper, Synthesis and characterization of novel bis-triazenes:  3,8-di[2-aryl-1-azenyl]-1,3,6,8-tetraazabicyclo[4.4.1]undecanes and 1,3-di-2-[(4-methoxyphenyl)-1-diazenyl]imidazolidine. The reaction of diazonium Ions with ethylenediamine/formaldehyde mixtures*,* *The Journal of Organic Chemistry* **1998**, *63*, 7437-7444.

[68] H. K. Paul Ehrlich, (Ed.: G. Brauer), Ferdinand Enke Verlag, Stuttgart, **1978**, pp. 1337–1341.

[69] J. L. B. Lygo, G. Procter, Praxis der organischen Chemie, Wiley-VCH, Weinheim, **1996**.

[70] W. I. F. David, K. Shankland, J. van de Streek, E. Pidcock, W. D. S. Motherwell, J. C. Cole, DASH: a program for crystal structure determination from powder diffraction data*,* *Journal of Applied Crystallography* **2006**, *39*, 910-915.

[71] C. J. McMonagle, D. R. Allan, M. R. Warren, K. V. Kamenev, G. F. Turner, S. A. Moggach, High-pressure sapphire capillary cell for synchrotron single-crystal X-ray diffraction measurements to 1500 bar*,* *Journal of Applied Crystallography* **2020**, *53*, 1519-1523.

[72] V. A. Yartys, R. V. Denys, J. P. Maehlen, C. Frommen, M. Fichtner, B. M. Bulychev, H. Emerich, Double-bridge bonding of aluminium and hydrogen in the crystal structure of γ-AlH_3_*,* *Inorganic Chemistry* **2007**, *46*, 1051-1055.

[73] A. A. Coelho, TOPAS and TOPAS-Academic: an optimization program integrating computer algebra and crystallographic objects written in C++*,* *Journal of Applied Crystallography* **2018**, *51*, 210-218.

[74] CCDC 2386632, CCDC 2386633, and CCDC 2386676 contain the supplementary crystallographic data for this paper. These data are provided free of charge by the joint Cambridge Crystallographic Data Centre and Fachinformationszentrum Karlsruhe Access Structures service.
